# Clinical outcomes during and after wearable cardioverter defibrillator use in Japanese patients with heart failure: A single‐center experience

**DOI:** 10.1002/joa3.13158

**Published:** 2024-10-04

**Authors:** Noriko Kikuchi, Tsuyoshi Shiga, Yohei Sugawara, Atsushi Suzuki, Yoshiaki Minami, Hidetoshi Hattori, Morio Shoda, Nobuhisa Hagiwara, Junichi Yamaguchi

**Affiliations:** ^1^ Department of Cardiology Tokyo Women's Medical University Tokyo Japan; ^2^ Department of Clinical Pharmacology and Therapeutics The Jikei University School of Medicine Tokyo Japan; ^3^ Clinical Research Division for Heart Rhythm Management Tokyo Women's Medical University Tokyo Japan

**Keywords:** heart failure, implantable cardioverter defibrillator, nonischemic, outcome, wearable cardioverter defibrillator

## Abstract

**Background:**

A wearable cardioverter defibrillator (WCD) is indicated for a limited period in patients at high risk of sudden cardiac death (SCD). Nonischemic heart failure (HF) is common among Japanese patients with HF. The aim of this study was to evaluate the incidence of fatal arrhythmias during WCD use and the clinical outcomes after WCD withdrawal in Japanese patients with HF.

**Methods:**

We retrospectively studied 105 hospitalized HF patients who were discharged with a WCD. The main outcome was SCD/ventricular arrhythmias during WCD use and the other outcomes were implantation of an implantable cardioverter‐defibrillator (ICD), SCD/ventricular arrhythmias after WCD withdrawal, and changes in left ventricular ejection fraction (LVEF).

**Results:**

Eighty‐seven (83%) patients received a WCD for primary prevention of SCD, of whom 60 (69%) were new‐onset HF patients with an LVEF ≤35%. The median daily wear time was 22.1 h. Two patients experienced sustained ventricular tachycardia and one patient experienced atrioventricular block with asystole while on WCD. After WCD withdrawal, 81 (77%) patients decided not to receive ICD implantation. The percentage of patients with an LVEF ≥35% increased from 20% at baseline to 70% at 1 year after discharge. During the median follow‐up of 50 months, 78 (96%) of the 81 patients who did not have an ICD were free of SCD/ventricular arrhythmias.

**Conclusions:**

The use of a WCD is useful for determining the appropriate indication for ICD implantation in Japanese patients with new‐onset HF, a low LVEF, and a risk of SCD.

## Graphical abstract

Among 105 patients with HF who were prescribed a WCD, two patients experienced sustained VT and one patient experienced AV block while on a WCD. Only 17 patients subsequently received an ICD.

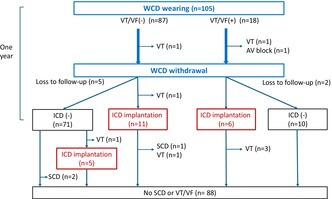

## 1 INTRODUCTION

Sudden cardiac death (SCD) is the leading cause of cardiovascular death in patients with heart failure (HF).^1^ Implantable cardioverter defibrillators (ICDs) are known to reduce SCD in patients with systolic HF or previous myocardial infarction.^2^, ^3^, ^4^ However, ICDs are associated with harm, including inappropriate shocks, device infection, and psychological distress.^5^

Optimal medical therapy (OMT) and interventions such as revascularization can improve the left ventricular ejection fraction (LVEF) and clinical outcome within several months in patients with new‐onset systolic HF or myocardial infarction.^6^, ^7^, ^8^, ^9^ We previously reported a higher incidence of SCD and ventricular tachyarrhythmia within 12 months of discharge in newly hospitalized systolic HF patients, and LVEF improvement was inversely associated with late SCD.^10^ However, patients remain at risk for SCD until left ventricular (LV) reverse remodeling and hemodynamic/HF symptoms stabilize. An observational study reported that improvement in LVEF was associated with a lower risk of all‐cause mortality and appropriate shocks in HF patients with ICDs for primary prevention of SCD.^11^

European and U.S. guidelines recommend a wearable cardioverter defibrillator (WCD) for patients who have a clear indication for an ICD but for whom an ICD is not indicated for temporary reasons, such as infection, or for patients at high risk of SCD in the early phase after myocardial infarction.^12^, ^13^ Because nonischemic HF is common among Japanese patients with HF,^14^ Japanese guidelines recommend the use of a WCD for a period of less than 3 months after initiation of pharmacological therapy or receiving revascularization, especially for new systolic nonischemic HF patients with LVEF ≤35% or ischemic HF patients with LVEF ≤35%, respectively.^15^ However, it should be noted that the recovery of LVEF, especially in nonischemic HF patients, exceeds 3 months, unlike that in ischemic HF patients.^10^, ^16^ Therefore, the aim of this study was to evaluate the incidence of SCD or sustained ventricular tachycardia (VT)/fibrillation (VF) during WCD use and the clinical outcomes after WCD use in Japanese patients with HF.

## 2 METHODS

### 2.1 Subjects

**FIGURE 1:**
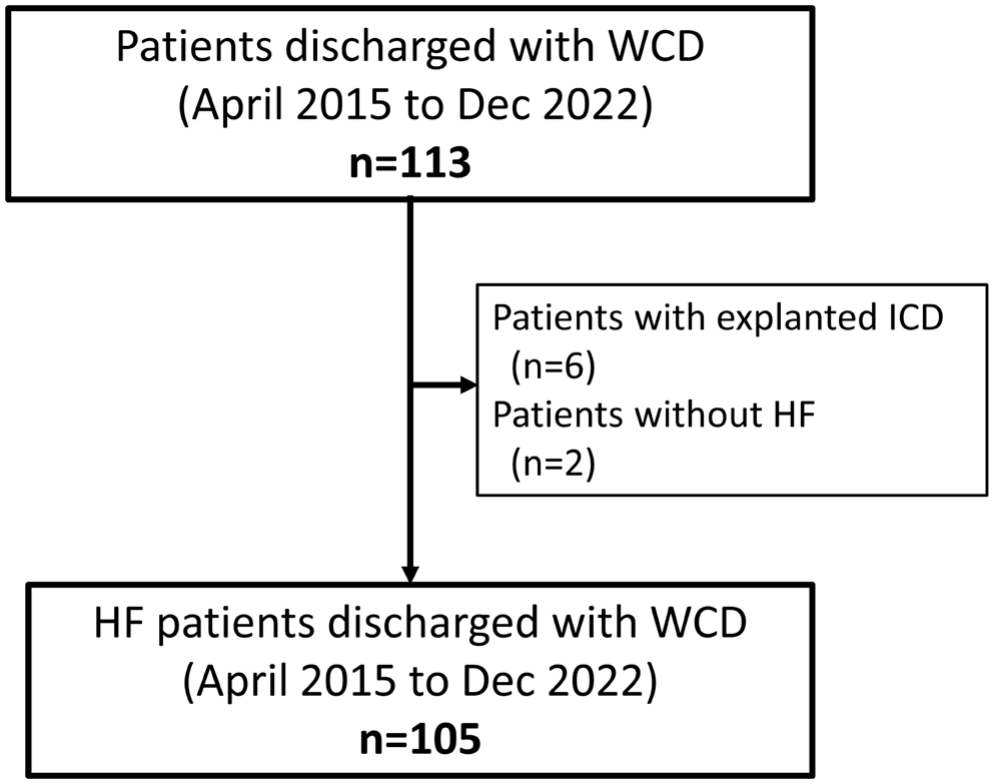
Patient flow chart for this study. HF, heart failure; WCD, wearable cardioverter‐defibrillator.

We retrospectively conducted an observational study of 113 consecutive hospitalized patients who were discharged with a WCD from the Department of Cardiology, Tokyo Women's Medical University Hospital between April 2015 and December 2022. WCD therapy was administered according to Japanese guidelines.^15^ We excluded 6 patients who had a WCD because of an explanted ICD due to device‐related problems such as infection or fracture. We also excluded 2 patients who did not have HF (1 patient with long QT syndrome and 1 patient with coronary spastic angina). Ultimately, we included 105 HF patients who received a WCD in the present study (Figure 1).

All patients received optimal therapy during hospitalization, including medical therapy, revascularization for coronary artery disease, catheter ablation for arrhythmias, and surgery. According to current HF guidelines, OMT for HF patients with reduced LVEF included angiotensin‐converting enzyme inhibitors/angiotensin II receptor blockers/angiotensin receptor neprilysin inhibitors (ACEIs/ARBs/ARNIs), beta‐blockers, and mineralocorticoid receptor antagonists.^17^, ^18^ This study was approved by the institutional review boards of Tokyo Women's Medical University (2020–0028).

### 2.2 WCD

In this study, Life Vest™ (ZOLL, Pittsburgh, USA) was used for all patients. Life Vest™ consists of a vest with electrocardiographic and defibrillation electrodes that continuously monitors and analyzes the patient's electrocardiogram to detect fatal arrhythmias. If a fatal arrhythmia, VT/VF, is detected, the monitor/battery belt automatically delivers an electric shock through the defibrillation electrodes within approximately 1 min after an audible alarm. The system and its programmed data have recently been described in detail.^19^ In most patients, the VT zone was programmed at a heart rate of 150 bpm with a VT response time of 60 s, and the VF response zone was programmed at a heart rate of 200 bpm with a VF response time of 25 s. The initial shock energy was set to the maximum power (150 J) in all patients. Each episode was reviewed by the Zoll Patient Management Network and classified into the following categories: sustained VT (lasting 30 s or longer) or VF with/without WCD shock therapy and asystole. Inappropriate WCD therapy was classified as a non‐VT/VF episode treated with WCD shock. Information on wearing days and hours per day was also collected from the Zoll Patient Management Network.

### 2.3 Outcomes

The primary outcome was a composite of SCD, sustained VT, or VF during WCD use; SCD was defined as nontraumatic unexpected death within 1 h of symptom onset or within 24 h of the last known time alive if the death was not witnessed due to specific circulatory failure. The secondary outcome was implantation of an ICD within 12 months of WCD withdrawal; the decision to implant an ICD was left to the discretion of each attending physician with patient consent according to Japanese guidelines; it is recommended that an ICD be implanted if the LVEF remains <35% after 3 months of OMT and if the patient has symptomatic HF. If the patient received an appropriate WCD shock, the ICD was implanted as secondary prophylaxis. The other outcome was the occurrence of VT/VF, which included appropriate ICD therapy for VT/VF after WCD withdrawal. In addition, changes in LVEF were assessed by echocardiography, and LVEF improvement was defined as LVEF ≥35% in this study.

### 2.4 Data collection

Patient information regarding demographic data, baseline clinical characteristics, underlying cardiac disease, laboratory data, treatment during hospitalization, and clinical course after discharge was collected from the electronic medical records. Coronary artery disease was defined as positive stress test findings, coronary angiography demonstrating at least 75% stenosis or coronary spastic angina documented by an acetylcholine provocation test, a history of prior myocardial infarction, or a history of revascularization procedures. Ischemic cardiomyopathy was defined as LV systolic dysfunction due to coronary artery disease. Nonischemic cardiomyopathy was defined as structural and functional ventricular abnormalities in the absence of coronary artery disease. Acute myocardial infarction was defined according to the fourth universal definition.^20^

WCD therapy could be covered for up to 3 months with Japanese medical insurance coverage. Most patients were scheduled to use the WCD for 3 months, but the timing of WCD withdrawal was left to the discretion of each attending physician. While wearing the WCD, the patients were followed up once per month, and information regarding the WCD was obtained from the Zoll Patient Management Network. During this time, HF therapy was intensified, and the indication for ICD was comprehensively determined based on follow‐up echocardiography and HF status at the time of WCD withdrawal. Follow‐up data after WCD withdrawal were obtained during routine or ad hoc visits to our outpatient clinic. Patients with ICDs were followed by telemonitoring and face‐to‐face visits to our ICD clinic every 3–6 months. Data on VT/VF events requiring ICD therapy, including both shock and antitachycardia pacing, were obtained by reviewing event details and electrograms stored on the ICD disk. Patients were followed until death from any cause, loss to follow‐up, or December 2023. Information on deceased patients was obtained from medical records, family members, general practitioners, and the hospital where they were admitted. Seven patients were lost to follow‐up within 12 months after discharge because they were subsequently referred to other hospitals after WCD withdrawal. These patients were excluded from the evaluation of long‐term outcomes in the present study.

### 2.5 Echocardiography

Echocardiography was performed by experienced sonographers using SONOS 5500/iE33/EPIQ7 (Philips Healthcare, Andover, Massachusetts, USA), ARTIDA (Toshiba Medical Systems, Tochigi, Japan), or GE Vivid E9/Vivid 7 (GE Vingmed Ultrasound AS, Horten, Norway) during hospitalization, 3 months after discharge, and 6–12 months after index hospital discharge. Echocardiographic data were measured by independent investigators who were blinded to the patient data; LV end‐diastolic volume and LV end‐systolic volume were measured in the apical two‐chamber view and four‐chamber view. From these results, the LVEF was calculated using the biplane Simpson method.

### 2.6 Statistical analysis

The summary data are presented as the number of patients or as the median and interquartile range. Univariate and multivariate Cox regression analyses were used to estimate the relationships between baseline clinical characteristics and an improved LVEF >35%. Clinical variables were selected based on previously reported predictors of clinical outcomes, such as age <60 years, estimated glomerular filtration rate ≥60 mL/min/1.73 m^2^, plasma brain natriuretic peptide level < 170 pg./mL,^21^, ^22^ nonischemic etiology, use of beta‐blockers, use of ACEIs/ARBs/ARNIs, use of cardiac resynchronizing therapy, and electrocardiographic and echocardiographic parameters. Multivariate analysis was performed using a forward stepwise method, with entry or removal based on a *p* value of <.10. Longitudinal changes in LVEF over time were tested using Friedman's test. Cumulative event‐free rates were calculated using the Kaplan–Meier method and compared using the log‐rank test. A p value <.05 was considered to indicate statistical significance. Data analysis was performed using IBM SPSS Statistics (version 29.0.0.0, IBM Corp., Armonk, NY, USA).

## 3 RESULTS

### 3.1 Baseline characteristics

Between April 2015 and December 2022, 105 patients were prescribed a WCD. The baseline characteristics of the patients are shown in Table 1. The median age was 55 years, and the proportion of men was 88%. Among these patients, nonischemic cardiomyopathy, including idiopathic dilated cardiomyopathy, was the most common heart disease, followed by ischemic cardiomyopathy. Ten percent of patients had acute myocardial infarction. Hemodialysis was performed in 6 (6%) patients. Nonsustained VT was detected in 66% of patients during hospitalization. The median LVEF was 29% and 80% of the patients had an LVEF ≤35%. Gadolinium‐enhanced cardiac magnetic resonance (CMR) was performed within 6 months of the index hospitalization in 60 patients, of whom 48 (80%) had late gadolinium enhancement (LGE).

**TABLE 1:** Baseline characteristics (*n* = 105).

Age	55 [49—65]
Male	89 (88%)
Body mass index (kg/m^2^)	24.3 [22.0—27.1]
Underlying heart diseases	
Ischemic cardiomyopathy	21
Acute myocardial infarction	10
Idiopathic dilated cardiomyopathy	42
Arrhythmia‐induced cardiomyopathy	11
Cardiac sarcoidosis	9
Arrhythmogenic right ventricular cardiomyopathy	1
Secondary cardiomyopathy	2
Valvular heart disease	6
Congenital heart disease	2
Acute myocarditis	1
NYHA functional class III/IV at admission	66 (63%)
Plasma BNP level at admission (pg/mL)	420 [125—1029]
eGFR (mL/min/1.73m^2^)	56.8 [48.0—74.2]
Comorbidities	
Hypertension	48 (46%)
Diabetes mellitus	39 (37%)
Dyslipidemia	45 (43%)
CRF undergoing hemodialysis	6 (6%)
Persistent atrial fibrillation	20 (19%)
Sustained VT/VF	18 (17%)
Nonsustained VT	69 (66%)
Electrocardiographic parameters	
Heart rate (bpm)	90 [72—101]
QRS duration (ms)	104 [92—124]
QTc (ms)	432 [413—454]
Echocardiography	
LVEDD (mm)	60 [52—66]
LVESD (mm)	54 [43—60]
LVEF (%)	29 [22—34]
Cardiac magnetic resonance (n=60)	
Late gadolinium enhancement	48 (80%)
Invasive treatment during hospitalization	
PCI	19 (18%)
CABG	6 (6%)
Other cardiac surgeries	5 (5%)
Catheter ablation	12 (11%)
Medications at discharge	
ACE inhibitors/ARBs/ARNIs	100 (95%)
Beta blockers	100 (95%)
MRAs	84 (80%)
SGLT2 inhibitors	23 (22%)
Digoxin	9 (9%)
Amiodarone	45 (43%)

Note: Values are presented as numbers (%) or medians (interquartile ranges).

Abbreviations: ACE, angiotensin‐converting enzyme; ARB, angiotensin II receptor blocker; ARNIs, angiotensin receptor neprilysin inhibitors; BNP, brain natriuretic peptide; CABG, coronary artery bypass graft; CRF, chronic renal failure; eGFR, estimated glomerular filtration rate; LVEDD, left ventricular end‐diastolic dimension; LVEF, left ventricular ejection fraction; LVESD, left ventricular end‐systolic dimension; MRA, mineralocorticoid receptor antagonist; NYHA, New York Heart Association; PCI, percutaneous coronary intervention; QTc, corrected QT interval; SGLT, sodium glucose cotransporter, VF, ventricular fibrillation; VT, ventricular tachycardia.

All patients received optimal therapy during hospitalization. Most patients were prescribed beta‐blockers and ACEIs/ARBs/ARNIs. Twenty‐four percent of patients underwent revascularization for coronary artery disease and 18% underwent cardioversion or catheter ablation for atrial tachyarrhythmias.

### 3.2 Indications for WCD therapy

**FIGURE 2:**
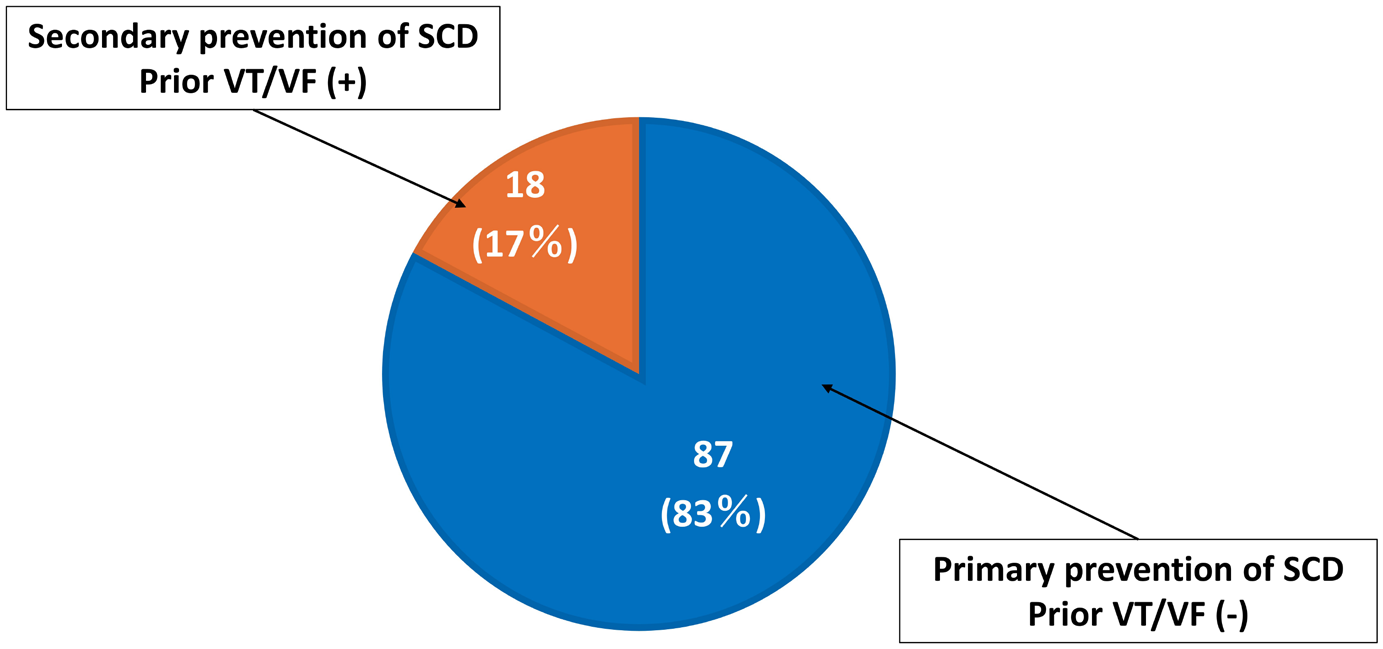
Primary and secondary prevention of SCD in HF patients wearing a WCD. HF, heart failure; SCD, sudden cardiac death; VF, ventricular fibrillation; VT, ventricular tachycardia; WCD, wearable cardioverter‐defibrillator.

Eighteen patients (17%) who had a history of VT/VF received WCD. The remaining 87 patients (83%) received a WCD for primary prevention of SCD (Figure 2). The reasons for WCD therapy in patients with a history of VT/VF were acute conditions such as acute myocardial infarction (*n* = 8), acute myocarditis (*n* = 1), evaluation of etiology and follow‐up of LVEF immediately after catheter ablation (*n* = 3) or cardiac surgery (n = 1), inducible VT during transesophageal echocardiography (n = 1) and refusal of ICD implantation (n = 1). Three patients wore a WCD during etiology evaluation or while awaiting surgery. Two patients experienced sustained VT late after repair of tetralogy of Fallot. After successful ablation for stable sustained VT, pulmonary valve replacement was planned for moderate pulmonary valve regurgitation, and the patients were discharged with a WCD. The ICD implantation was scheduled after pulmonary valve replacement. Another patient presented with suspected cardiac sarcoidosis and sustained VT. The patient was discharged with a WCD for an outpatient positron emission tomography (PET) scan; PET confirmed disease activity, and the ICD was subsequently implanted.

Among patients with no history of VT/VF who received a WCD for the primary prevention of SCD, most had a low LVEF and/or newly diagnosed HF.

### 3.3 Arrhythmia events during WCD use

Information on WCD wearing and arrhythmic events during WCD use is shown in Table 2. The median daily WCD wear time was 22.1 h, and 19 (18%) of the patients had a daily wear time of less than 15 h. The median number of wear days was 65 days. No patient experienced SCD during WCD use. VT/VF during WCD use occurred in 2 patients (2%). In one patient with ischemic cardiomyopathy, the WCD delivered appropriate shocks for sustained unstable VT but this action was primary prevention. In another patient with congenital heart disease (Tetralogy of Fallot), sustained VT was detected, but her hemodynamics were stable, and the shock delivery was stopped. In addition, paroxysmal complete AV block with ventricular asystole was detected in one patient with myocarditis. All patients subsequently underwent ICD implantation.

**TABLE 2:** WCD wearing information and arrhythmic events during WCD use.

Wearing information	
Wearing days	65 [34—80]
Daily wearing time (h)	22.1 [17.3—23.1]
Arrhythmic events	
Sustained VT/VF	2 (2%)
AV block with asystole	1 (1%)
WCD therapies	
Appropriate shock	1 (1%)
Inappropriate shock	0

*Note*: Values are presented as numbers (%) or medians (interquartile ranges).

Abbreviations: AV, atrial‐ventricular; VF, ventricular fibrillation; VT, ventricular tachycardia; WCD, wearable cardioverter defibrillator.

### 3.4 ICD implantation after WCD withdrawal

The status of patients who underwent ICD implantation in the first year after discharge following WCD withdrawal is shown in Table 3. Seventeen patients (16%) underwent ICD implantation, including 6 patients who received cardiac resynchronization therapy with an ICD. Nine patients underwent ICD implantation for primary prevention: one patient with inducible VT on electrophysiological study after WCD withdrawal and eight other patients with persistent low LVEF and HF symptoms (NYHA functional class II/III). The common reasons for not implanting ICDs were improvement in LVEF and improvement in HF status (NYHA functional class I). Despite having indications for ICD implantation, 3 patients refused this treatment.

**TABLE 3:** ICD implantation 1 year after discharge following WCD withdrawal.

ICD implantation	17 (16%)
Secondary prevention indication	8
Primary prevention indication	9
No ICD	81 (77%)
Reasons for not implanting ICDs	
Improved LVEF	49
NYHA functional class I	28
Patient refusal	3
LVAD implantation	1
Unknown	7 (7%)

*Note*: The values are numbers (%).

Abbreviations: ICD, implanted cardioverter defibrillator; LVAD, left ventricular assist device; LVEF, left ventricular ejection fraction; NYHA, New York Heart Association.

### 3.5 Changes in LVEF in patients with WCD

**FIGURE 3:**
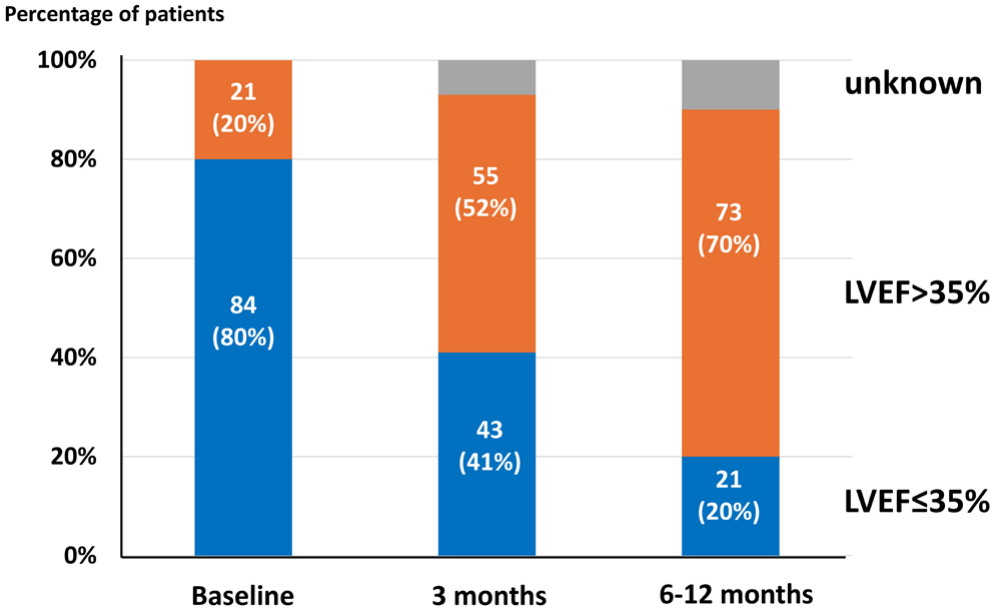
Follow‐up LVEF in HF patients wearing a WCD. HF, heart failure; LVEF, left ventricular ejection fraction; WCD, wearable cardioverter‐defibrillator.

Longitudinal changes in LVEF in patients with WCD were as follows: median LVEF of 29^7^, ^8^, ^22^, ^23^, ^24^, ^25^, ^26^, ^27^, ^28^, ^29^, ^30^, ^31^, ^32^ % at baseline, 38 [31–48] % at 3 months, and 46 [37–55] % at 6–12 months (*p* < .001). Eighty‐four (80%) patients had an LVEF ≤35% on admission. Although 43 (41%) patients still had an LVEF ≤35% at 3 months after hospital discharge, the remaining patients had an LVEF >35%. In addition, follow‐up echocardiography at 6–12 months after discharge revealed that only 21 patients had an LVEF ≤35%, with approximately 70% of patients showing an LVEF >35% (Figure 3). Multivariate analysis revealed that a QRS duration <130 msec and an LV end‐diastolic dimension <60 mm were predictors of LVEF improvement >35% (Table 4).

**TABLE 4:** Factors associated with LVEF >35% at 6–12 months.

Variable	Univariate analysis			Multivariate analysis		
	OR	95% CI	*p* value	OR	95% CI	*p* value
Age <60 years	1.14	0.42–3.09	.80			
Nonischemic etiology	2.17	0.79–5.96	.13			
New‐onset HF	4.71	1.67–13.28	<.01	3.75	0.95–11.88	.06
Baseline LVEF >30%	9.00	1.96–41.43	.01	5.69	0.83–38.95	.08
Baseline LVEDD <60 mm	21.11	2.69–165.55	<.01	13.44	1.42–127.01	.02
Baseline QRS duration <130 msec	2.89	1.00–8.38	.05	7.52	1.53–37.05	.01
Heart rate <75 bpm	0.85	0.30–2.38	.75			
Plasm BNP <170 pg/mL at discharge	1.11	0.41–3.03	.84			
eGFR ≥60 mL/min/1.73 m^2^	1.20	0.45–3.18	.71			
Use of beta‐blockers	3.79	0.50–28.68	.20			
Use of ACEIs/ARBs/ARNIs	6.00	0.93–38.83	.06	3.48	0.28–43.82	.33

Abbreviations: ACEIs, angiotensin‐converting enzyme inhibitors; ARBs, angiotensin II receptor blockers; ARNIs, angiotensin receptor neprilysin inhibitors; BNP, brain natriuretic peptide; CI, confidence interval; CRT, cardiac resynchronization therapy; eGFR, estimated glomerular filtration rate; OR, odds ratio; LVEDD, left ventricular end‐diastolic dimension; LVEF, left ventricular ejection fraction.

### 3.6 Outcomes after WCD withdrawal

**FIGURE 4:**
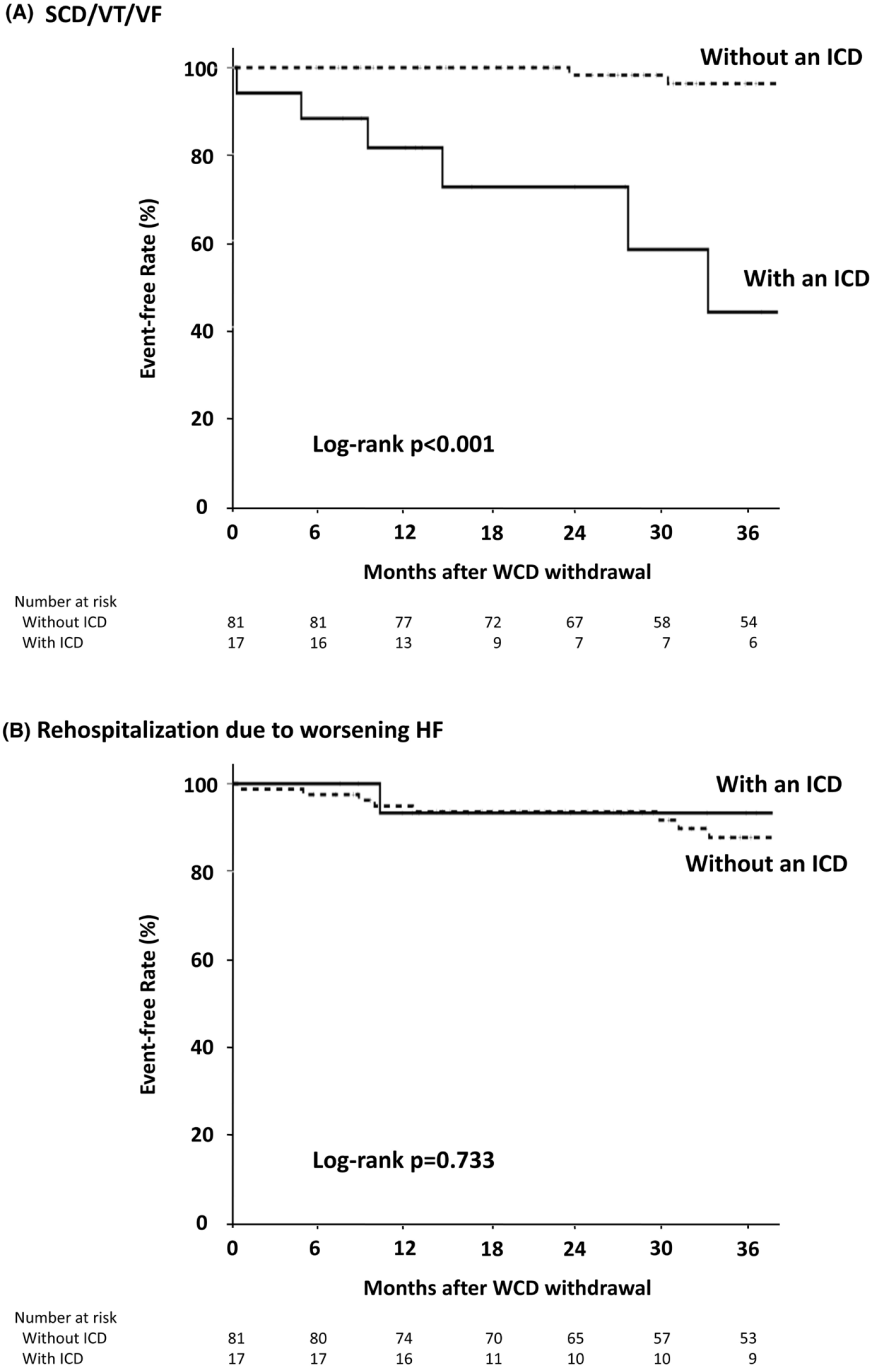
SCD/arrhythmic events (A) and rehospitalization due to worsening HF (B) in patients with or without an ICD. HF, heart failure; ICD, implantable cardioverter defibrillator; SCD, sudden cardiac death; VF, ventricular fibrillation; VT, ventricular tachycardia.

During a median follow‐up of 50 [32–69] months, 9 patients experienced SCD or VT/VF, and 11 patients were rehospitalized due to worsening HF. The incidence of SCD or sustained VT/VF (including appropriate ICD therapy) after WCD withdrawal was greater in patients who underwent ICD implantation in the first year after discharge than in those who did not (Figure 4(A)).

Among 17 patients who underwent ICD implantation in the first year after discharge, 1 patient experienced SCD, and 4 patients were successfully treated with ICD therapies for VT/VF. One SCD patient was a hemodialysis patient with ischemic cardiomyopathy and an LVEF of 38%. After WCD withdrawal, the patient was implanted with an ICD because of inducible sustained VT in an electrophysiological study. Approximately 3 years after discharge, he died suddenly due to VF at home; ICD shocks failed to terminate the VF.

In contrast, among the 81 patients who did not receive an ICD in the first year after discharge, two experienced SCD, and one experienced sustained VT requiring external direct current cardioversion for termination. These two SCD patients were hemodialysis patients. One SCD patient underwent coronary artery bypass grafting for ischemic cardiomyopathy, and his HF symptoms improved (NYHA functional class I), but his LVEF did not improve. An electrophysiological study was not performed. Another SCD patient underwent percutaneous coronary intervention for ischemic cardiomyopathy, and his LVEF improved from 34% to 50% but decreased to 20% 3 years later; the patient subsequently experienced SCD while an ICD implantation was being considered. One patient with sustained VT received subsequent ICD implantation. In the remaining 78 (96%) patients who did not receive an ICD in the first year after discharge, no fatal ventricular arrhythmias occurred during the follow‐up.

The incidence of rehospitalization for worsening HF did not differ between patients with and without ICDs after WCD withdrawal. (Figure 4(B)).

## 4 DISCUSSION

In this study, we evaluated the clinical characteristics and arrhythmic outcomes of 105 patients with HF who were prescribed a WCD at discharge from our hospital and found the following: (1) 87 (83%) patients received a WCD for primary prevention of SCD, of whom 60 (69%) had newly diagnosed HF with an LVEF ≤35%. (2) None of these patients experienced SCD, but 2 patients (2%) experienced sustained VT while using a WCD. (3) After WCD withdrawal, 81 (77%) patients decided not to receive ICD implantation. (4) The percentage of patients with an LVEF ≥35% increased from 20% at baseline to 70% one year after discharge. (5) In 96% of patients who did not receive an ICD after WCD withdrawal, no SCD or ventricular arrhythmia events occurred during the follow‐up.

### 4.1 WCD therapy in patients with newly diagnosed HF

In this observational study, approximately 70% of patients who underwent WCD treatment for primary prevention of SCD were newly diagnosed with HF with an LVEF ≤35%, and most of whom had nonischemic HF. The use of a WCD for the short‐term prevention of SCD was appropriate because of the risk of SCD early after HF treatment initiation but the possibility of long‐term improvement in LVEF.^8^, ^10^ In a previous report examining the risk of appropriate WCD therapy by etiology in patients with newly diagnosed cardiomyopathy, the frequency of ischemic cardiomyopathy patients receiving an appropriate shock during a median wearing period of 61 days was 2.2%, but none of the nonischemic cardiomyopathy patients received appropriate shock with WCD.^23^ In our study, one of 21 patients with ischemic cardiomyopathy received an appropriate shock while wearing a WCD.

A meta‐analysis of 28 studies reported a greater frequency of appropriate WCD therapy in patients with ischemic cardiomyopathy than in those with nonischemic cardiomyopathy (8 vs. 6 per 100 persons over 3 months, respectively); four of the 8 studies of patients with nonischemic cardiomyopathy reported an event rate of 0 [24.] In our study, none of the patients with nonischemic cardiomyopathy received appropriate WCD therapy. However, this finding may be due to the sample size. Our previous study revealed that 4 (3%) of 125 patients with newly diagnosed nonischemic cardiomyopathy developed SCD or VT within 3 months after hospital discharge.^10^ Patients with ischemic cardiomyopathy are less likely to improve their LVEF despite surgical or medical therapy, which is also associated with mortality.^25^ This issue may contribute to the high incidence of ventricular arrhythmias in patients with ischemic cardiomyopathy.

However, the Vest Prevention of Early Sudden Death Trial (VEST) revealed that a WCD did not significantly reduce the incidence of arrhythmic death in patients with acute myocardial infarction and a low LVEF compared with the corresponding incidence in controls.^26^ Nevertheless, WCD use significantly reduced the incidence of arrhythmic death in the as‐treated and per‐protocol analyses, partly due to compliance issues.^27^ Therefore, the use of a WCD benefits acute myocardial infarction patients at risk of SCD.

### 4.2 Adherence to WCD use

In this study, the median daily wear time of WCD was 22 h; however, 18% of the patients had a daily wear time of less than 15 h, which is considered very low adherence.^26^ This wear time is comparable to the wear time reported in previous observational studies (20–23.5 h per day).^24^, ^28^ Fortunately, no patient in this study experienced an arrhythmic event during WCD removal. A subanalysis of VEST data revealed that deaths, including arrhythmic deaths, were less common when a WCD was worn than when it was not worn.^27^ Asians and younger people are also reported to be at risk for nonadherence to WCD.^27^, ^29^ In this study, the median wear duration was 58 days, that is, less than 90 days. A WCD reduces a patient quality of life due to discomfort, fear of shock, and behavioral limitations, which leads to worsened adherence and early discontinuation.^30^ To improve adherence, the purpose of the WCD needs to be explained to patients, standardized education on its use needs to be provided, and adherence needs to be monitored and managed.^30^

The duration of WCD treatment remains controversial. The LVEF may not fully improve during this period, particularly in patients with nonischemic cardiomyopathy, and in this study, one patient experienced sustained VT immediately after WCD withdrawal. However, in view of the distress that a WCD causes patient, simply extending the number of wear days is not advisable. In this study, even if WCD use was limited to 3 months, a decision could be made to implant a subsequent ICD.

### 4.3 LVEF and ventricular arrhythmia

In this study, the percentage of patients with an LVEF ≤35% at enrollment decreased from 80% to 40% at 3 months and further decreased to 20% at 6–12 months. These findings suggest that improvement in LVEF may reduce the risk of SCD and prevent ICD implantation. Furthermore, 96% of patients who did not have an ICD implanted within 12 months did not experience a fatal arrhythmic event during follow‐up. However, patients with no LVEF improvement at 6–12 months had a relatively high arrhythmic event rate (14%), and 24% were rehospitalized for worsening HF and were considered for ICD implantation. In addition, this study revealed that a narrow QRS duration (<130 msec) and mildly dilated LV end‐diastolic dimension (<60 mm) were independent predictors of an LVEF >35% at 6–12 months. A shorter QRS duration and smaller LV end‐diastolic dimension are known to improve within a few months with OMT in patients with new‐onset systolic HF.^7^, ^8^ Our previous study revealed that a narrow QRS duration (< 130 msec) was also an independent predictor of improved LVEF to >35% in patients with new‐onset systolic HF.^10^ A prolonged QRS duration has been reported to be associated with increased postdischarge morbidity and mortality in hospitalized HF patients with a low LVEF.^31^ In HF patients with a low LVEF, a narrow QRS duration may be an important marker of LVEF improvement and subsequent beneficial outcomes with optimal HF treatment.

The prevention of SCD with a WCD is a good strategy until HF is stabilized and the LVEF improves with intensified OMT for HF. Even patients whose LVEF improves and who avoid ICD implantation should be closely monitored for subsequent changes in their LVEF and HF status. If LVEF declines or HF worsens, the indication for an ICD should be re‐evaluated. In addition, with the recent introduction of new HF medications, further reductions in events and improvements in EF are expected.

### 4.4 SCD in hemodialysis patients

In this study, three patients experienced SCD after WCD withdrawal. All three patients were on hemodialysis. Hemodialysis patients are known to be at high risk for SCD,^32^, ^33^ and the pathophysiology of such SCD differs from that of the general population. The role of ICDs in hemodialysis patients is unclear.^34^ The pathophysiology of SCD may be related to vascular calcification and left ventricular hypertrophy, whereas traditional cardiovascular risk factors seem to have a more attenuated effect.^35^ In addition, electrolyte abnormalities, sympathetic overactivity, inflammation, etc. may also contribute to the occurrence of SCD.^36^ In our study, a hemodialysis patient with an ICD received shock therapy for VF; however, these shocks failed to terminate the VF, resulting in SCD (arrhythmic death). The Implantable Cardioverter‐Defibrillator in Dialysis Patients (ICD2) trial, a prospective, randomized, controlled trial, revealed that the incidence of SCD remains high in hemodialysis patients and that prophylactic ICD therapy does not reduce SCD.^37^

### 4.5 LGE on CMR and ventricular arrhythmia

Recently, LGE on CMR has been reported to be associated with increased ventricular arrhythmias and SCD in ischemic and nonischemic cardiomyopathies.^38^, ^39^ In this study, 57% of the eligible patients had undergone CMR and 80% of them had LGE. However, among patients who underwent CMR and were followed for more than 1 year, the occurrence of SCD or VT/VF during or after WCD wear did not significantly differ between patients with and without LGE (5/45 vs. 0/12, chi‐square *p* = .227). Because of the small number of subjects in this study, conclusions could not be drawn from these findings. Further evaluation with a large number of patients is needed to confirm this issue.

### 4.6 Study limitations

A retrospective, observational design was used in this study, which was conducted at a single center. Consecutively treated patients were enrolled in the study to minimize selection bias. Sixty‐seven (64%) patients in the study were hospitalized before 2020, the year in which ARNIs and sodium‐glucose cotransporter 2 inhibitors were approved for HF treatment in Japan. Therefore, HF drug therapy may have been subject to bias. Furthermore, the number of subjects was relatively small; therefore, a subgroup analysis was not feasible.

## 5 CONCLUSIONS

This study demonstrated that the majority of Japanese patients who received a WCD had newly diagnosed HF and a low LVEF, mostly of nonischemic etiology. In addition, 77% of them did not receive an ICD after WCD withdrawal, primarily due to improved LVEF or HF status with optimal therapy. The use of a WCD is useful for determining the appropriate indication for ICD implantation in Japanese patients with new‐onset HF, a low LVEF, and a low risk of SCD.

## FUNDING INFORMATION

Not applicable.

## CONFLICT OF INTEREST STATEMENT

Morio Shoda is an endowed chairperson of Biotronik, Boston Scientific, Medtronic, and St. Jude Medical. The remaining authors have no conflicts of interest to declare.

## ETHICS STATEMENT

The protocol was approved by the institutional review board of Tokyo Women's Medical University (2020–0028). IRB approval date: 2024/2/26.

## PATIENT CONSENT STATEMENT

The requirement to obtain written and verbal informed consent was waived because of the retrospective nature of the study. The study received ethical approval for the use of an opt‐out approach to obtain consent.

## CLINICAL TRIAL REGISTRATION

Not applicable

## STATEMENTS RELATING TO OUR ETHICS AND INTEGRITY POLICIES

This study was conducted in compliance with the Ethical Guidelines for Medical and Biological Research Involving Human Subjects issued by the Japanese Ministry of Health, Labour and Welfare and the Declaration of Helsinki.

## Data Availability

The data sets generated and/or analyzed during the current study are not publicly available due to privacy or ethical restrictions but are available from the corresponding author upon reasonable request.
